# Keratosis obturans: A rare cause of facial nerve palsy

**DOI:** 10.1002/ccr3.5410

**Published:** 2022-02-07

**Authors:** Prasanta Poudyal, Gaurav Nepal, Sushil Kumar Yadav, Yogesh Neupane, Heempali Dutta, Shishir Pokhrel, Prabin Gaire

**Affiliations:** ^1^ Department of Otorhinolaryngology‐Head and Neck Surgery Tribhuvan University Teaching Hospital Kathmandu Nepal; ^2^ Department of Internal Medicine Tribhuvan University Teaching Hospital Kathmandu Nepal; ^3^ Maharajgunj Medical Campus Tribhuvan University Institute of Medicine Kathmandu Nepal; ^4^ Department of Pathology Maharajgunj Medical Campus Tribhuvan University Institute of Medicine Kathmandu Nepal

**Keywords:** facial nerve palsy, facial palsy, keratin plug, keratosis obturans

## Abstract

Keratosis obturans, caused by the deposition of desquamated keratin plug in the external auditory canal can present with facial palsy. Young patients presenting with facial palsy, earache, and gradual hearing loss should be suspected for Keratosis obturans.

## Graphical abstract

Keratosis obturans, caused by the deposition of desquamated keratin plug in the external auditory canal, can present with facial palsy. Young patients presenting with facial palsy, earache, and gradual hearing loss should be suspected of keratosis obturans.

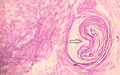

## 1 INTRODUCTION

Keratosis obturans (KO) is a rare disease of the external auditory canal affecting the age group <40 years. It is characterized by hearing loss and acute severe pain following the deposition of desquamated keratin plug in the external auditory canal.^1^ Before a case series published by Piepergerdes et al., keratosis obturans and external auditory canal cholesteatoma (EACC) were considered to be the single entity despite having different pathophysiology and clinical presentations.^2^ It has been suggested that the deposition of squamous debris is due to the fault in auditory epithelium migration and damage to the basal epithelial layer of the tympanic membrane.^3^

Keratosis obturans itself is a rare disease and rarely presents with facial nerve palsy, although other complications such as labyrinthine fistula, dehiscence of the tegmen, and dehiscence of the temporomandibular joint and jugular bulb have been reported.^4^ We report a case of keratosis obturans complicated with facial nerve palsy in a 13‐year‐old female patient. Before this, only six articles reporting the cases of KO with facial palsies are found.^2^, ^4^, ^5^, ^6^, ^7^, ^8^

## 2 CASE DESCRIPTION

A 13‐year‐old female patient presented to the outpatient of Otorhinolaryngology and Head and Neck Surgery of Tribhuvan University Teaching Hospital, Kathmandu, with chief complaints of left ear discharge for 4 years, decreased hearing in the left ear for 4 years followed by left earache for 15 days, and deviation of angle of mouth to the right side and inability to close the left eye for the same duration. Ear discharge was initially intermittent, however, continuous for the last 2 months, scanty, thick, and foul‐smelling. Hearing loss was insidious, progressive, non‐fluctuant, and not hampering her daily activities. The patient received treatment in another center with oral antibiotics, antibiotic ear drops, and analgesics. Documentation shows examination under a microscope being done there along with excision biopsy taken from aural polyp of the left external auditory canal; however, biopsy report was not available.

**FIGURE 1:**
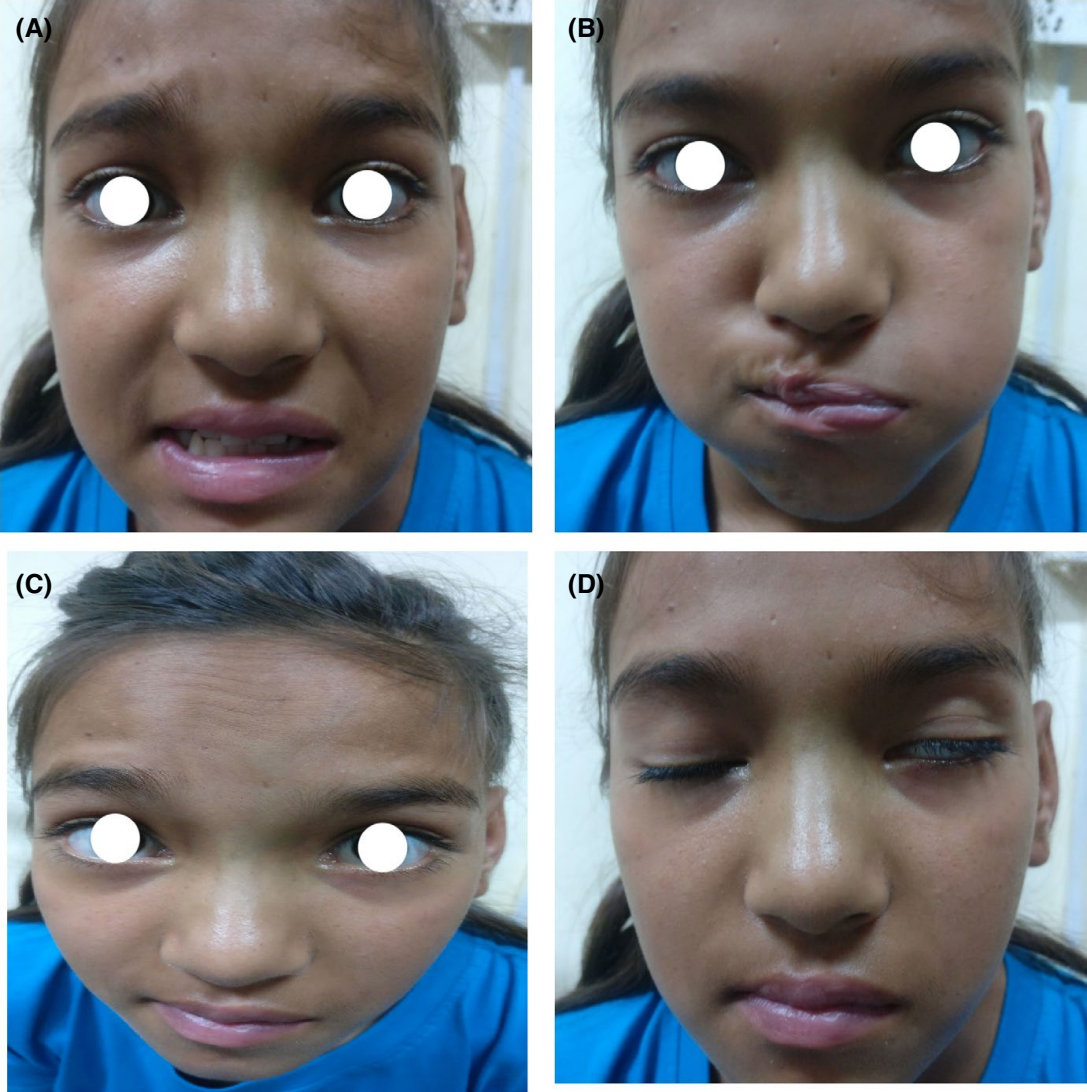
Examination of facial nerve shows (A) deviation of angle of mouth to right on smiling, (B) failure to blow the cheeks on the left, (C) loss of furrow in forehead on the left side, d‐incomplete closure of the left eye with maximal effort

On examination, the patient was comfortable but febrile. Facial nerve examination showed House‐Brackmann Grade III lower motor neuron palsy on the left side (Figure 1A‐C). There was left tragal and mastoid tenderness as well as tenderness on the various movement of the left pinna. Otoscopic examination revealed keratin debris with polypoidal tissue surrounded by foul‐smelling, yellowish mucopurulent discharge with narrowed cartilaginous external auditory canal (EAC) on the left side and hard wax on the right side. TM could not be visualized on either side. Tuning fork test showed Weber central with bilateral negative Rinne. Examination of the oral cavity, oropharynx, and nasal cavity was unremarkable, and neck nodes were not palpable. Vestibular tests were negative, and cerebellar signs were not elicited. All other cranial nerves were intact, and no other neurological deficit was evident. Systemic examination was normal. Neurosurgery consultation revealed no evidence of intracranial extension, and ophthalmology consultation revealed no papilledema.

**FIGURE 2:**
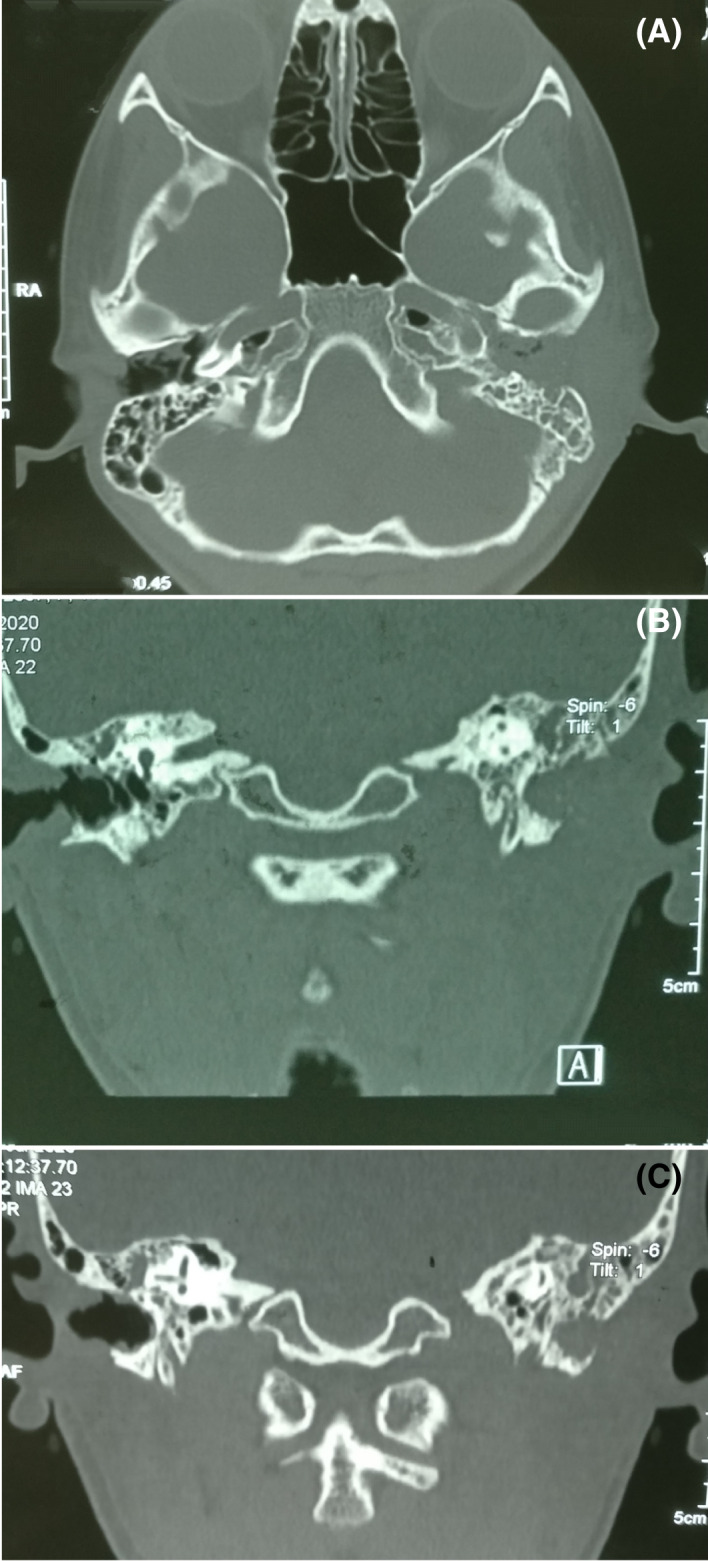
High‐resolution computed tomography (HRCT) scan of temporal bone revealing (A) ballooning of bony external auditory canal with destruction along with soft tissue density in mastoid, (B) soft tissue density in attic with facial canal dehiscence, (C) dural plate dehiscence

Complete blood counts and electrolytes were within the normal range. A pure tone audiogram done preoperatively showed bilateral moderate conductive hearing loss. High‐resolution computed tomography (HRCT) scan of temporal bone 0.6 mm cut axial view revealed ballooning of EAC with destruction, presence of soft tissue density in mastoid, and coronal view showed soft tissue density in non‐dependent part of the middle ear (attic), dehiscence of the facial canal, and dehiscence of the dural plate (Figure 2A‐C).

**FIGURE 3:**
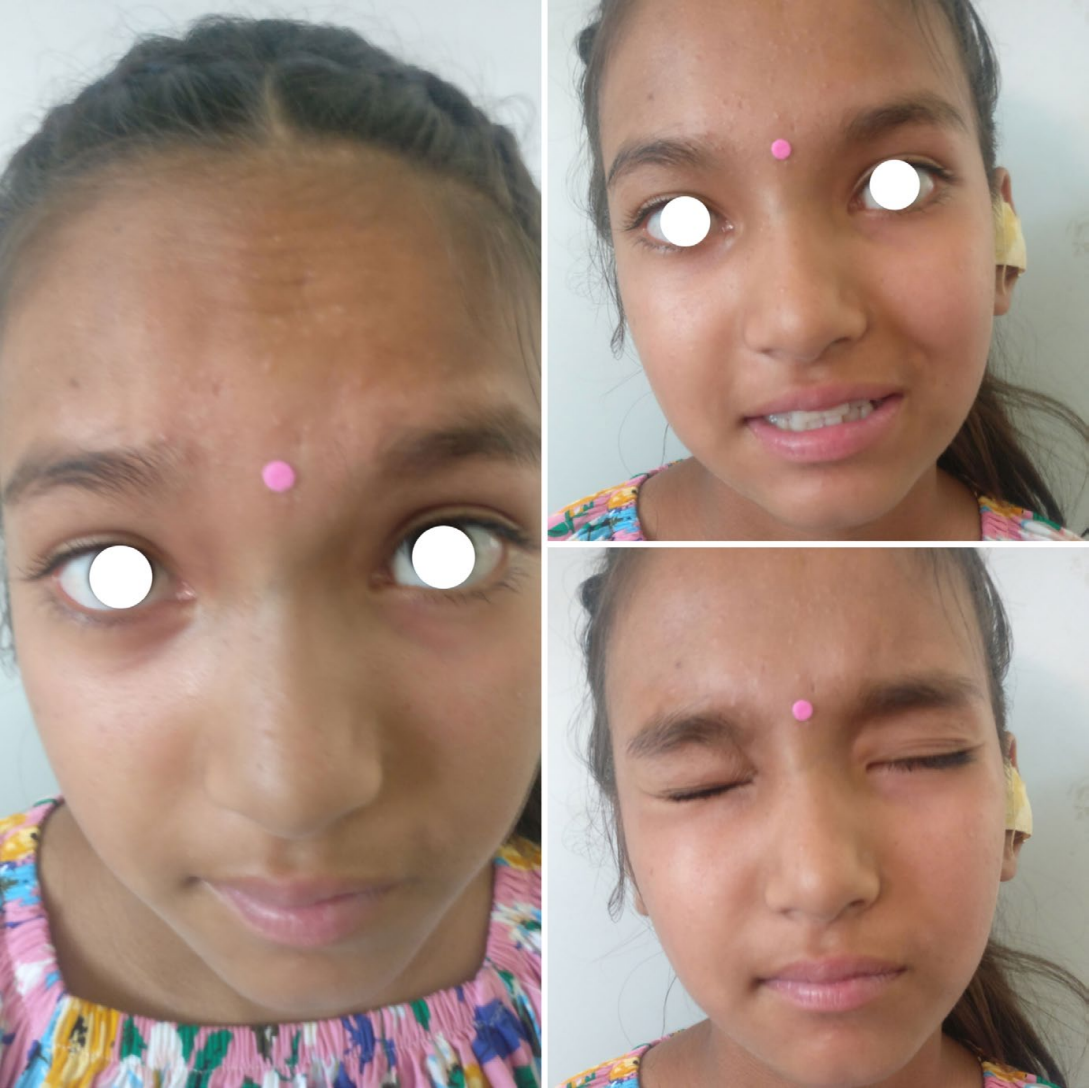
Follow‐up examination showing improvement of facial palsy

**FIGURE 4:**
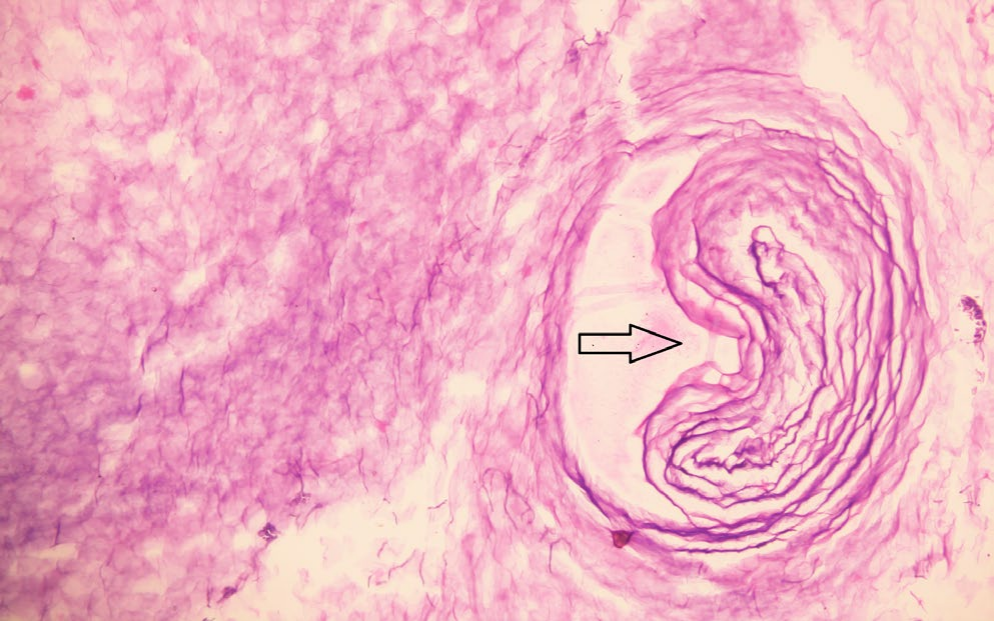
Histopathological examination (200×) of lesion showing keratin debris and keratin pearls (arrow)

**FIGURE 5:**
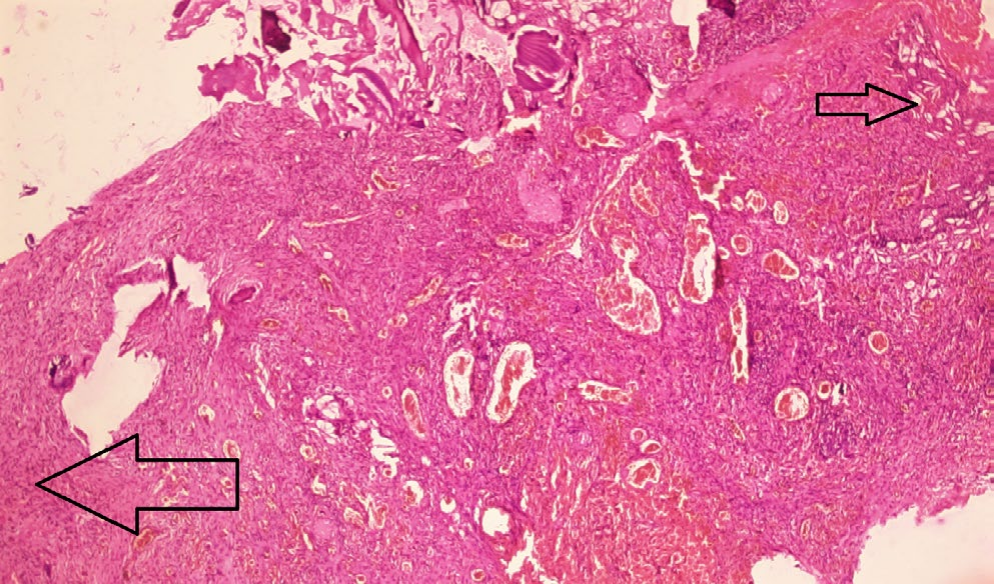
Histopathological examination (40×) of lesion showing cholesterol clefts (small arrow) and inflammatory cells (big arrow)

The patient was started on intravenous antibiotics, oral steroids, and analgesics. Preoperative examination under examination showed a narrow cartilaginous external auditory canal (EAC) with the circumferential widening of bony EAC, keratin debris with pus, and the tympanic membrane was missing on the diseased side. She underwent left modified radical mastoidectomy with myringostapediopexy. Intraoperatively, dehiscence of the tympanic segment of the facial nerve just posterior to cochleariformis process with intact sheath was found. Keratin debris was found in the attic, aditus, anterior part of antrum and mesotympanum, and squamous epithelium was found in attic and aditus. Antibiotics, steroids, and analgesics continued postoperatively and facial physiotherapy started, and the patient was followed up on an OPD basis after discharge on the 3rd postoperative day. On evaluation on the 10th postoperative day, there was a residual grade I left‐sided facial palsy only (Figure 3A‐C). Histopathology report showed polypoid tissue composed of granulation tissue with infiltration of dense mixed inflammatory cells containing plasma cells, lymphocytes, histiocytes, numerous cholesterol clefts, and keratin debris (Figures 4 and 5).

## 3 DISCUSSION

It is essential to differentiate KO from EACC to prevent misdiagnosis as they mimic each other clinically and treatment modalities are different for each.^2^ KO presents with keratin plug occluding EAC, generalized widening of EAC, thickening of TM, and hyperemia of the canal skin with granulations, while EACC presents with otorrhea, localized erosion of inferior EAC wall, and localized periostitis with sequestration of bone, but keratin plug is not found and the tympanic membrane remains normal.^1^ According to Piepergerdes et al., KO is mostly encountered in the age group <40 while EACC is encountered in the age group 45–75.^1^ However, the cases reported in the articles reviewed for this study show only 50% of patients are under 40 years of age, 22 years being the minimum.^2^, ^4^, ^5^, ^6^, ^7^, ^8^ Bony erosions in the cases of KO and EACC are different that is, it is extensive in the case of KO and relatively localized in the case of EACC.^1^ KO is bilateral in most pediatric cases while EACC is unilateral in most of the cases of all age groups.^1^ KO progresses to the middle ear via the tympanic membrane and the progression to the mastoid cavity in EACC occurs through the posterior EAC wall.^2^ Keratin plug in AO is lamellar, older layers lie centrally, and keratin is derived throughout EAC. While, in EACC, the only source of keratin is the sac of cholesteatoma, and keratin is spread randomly.^9^ KO is simply treated by the removal of keratin plug and, conservatively, while EACC requires surgical intervention. However, surgical intervention has been done in this case, even for KO, because of the involvement of the middle ear cavity and facial nerve.^2^

Faulty migration of the TM membrane's epithelium is suggested to be the etiologic basis of KO.^3^ In a normal person, epithelial migration occurs superiorly followed by posteriorly in zone I (epithelium of pars flaccida and malleus handle) and radially from malleus handle to the annulus in zone II (remaining pars tensa). The deposition of keratin on TM is associated with either no movement or slow movement in the abnormal pathway. The basal epithelial cells are assumed to get infected by the virus as KO is often accompanied by bronchiectasis and/or sinusitis. The inflammation of TM as a result of infection is supposed to initiate abnormal migration because basal epithelial cells are programmed for the migration. KO can also be of the silent type caused by the abnormal separation of keratin without inflammation and faulty migration of epithelium.^7^ This type of KO keeps recurring after the first removal of the keratin plug, therefore, requires continuous ear toileting.

Facial nerve palsy as a result of KO is very rare and is caused by bony erosion due to keratin plug pressure in EAC.^5^ When bony erosion is not depicted in HRCT temporal, as in this case, the facial nerve might be affected directly by pressure exerted by acute inflammation,^7^ because the resolution of facial palsy following removal of keratin plug and antibiotics administration is noticed. All the cases of KO with facial nerve palsy reported earlier had a year‐long history of ear symptoms, mostly gradual hearing loss. Exposed mastoid segment of the facial nerve, auto‐mastoidectomy, EAC granulation, and the aural polyp were present intraoperatively in previously reported cases,^6^ among which EAC granulation and the aural polyp are reported in this case as well. Despite conservative treatment after keratin plug removal being suggested, all the cases of KO with facial palsies were surgically managed and showed significant improvement. The case reported in this article has a four‐year‐long history of gradually progressive hearing loss and a prior history of aural polyp removal. After surgical removal of keratin plug, the patient has recovered from House‐Brackmann Grade III facial palsy to House‐Brackmann Grade I facial palsy in 10 postoperative days.

To rule out other causes of facial palsies, such as Bell's palsy, herpes zoster, otitis media, Lyme disease, Guillain–Barre syndrome, HIV infection, sarcoidosis, Sjogren syndrome, tumor, and stroke, detailed history and clinical examinations must be done followed by radiological and histological investigations. Patients developing ear symptoms associated with keratin deposition in EAC must be treated appropriately in time as KO, despite being a benign condition, can lead to severe complications as seen in this case. Ear toileting must be done continuously in patients with the recurrence of keratitis obturans.

## CONFLICT OF INTEREST

The authors declare that they have no competing interests.

## AUTHOR CONTRIBUTIONS

PP, SP, GN, HD, and YN were involved in patient care (diagnosis, treatment, and follow‐up). PG performed the histopathological examination of the specimen and provided us the required images. PP, GN, SY, and SP contributed to the collection of case information, writing of the manuscript, and manuscript revision. All authors approved the final version.

## ETHICAL APPROVAL

This study did not include experiments on animals or humans.

## CONSENT

Written informed consent was obtained from the patient's parents for the publication of this case report and any accompanying images. A copy of the written consent is available for review by the Editor‐in‐Chief of this journal.

## Data Availability

The data used in the case report are available on reasonable request.
